# *Spirulina platensis* Consumption Prevents Obesity and Improves the Deleterious Effects on Intestinal Reactivity in Rats Fed a Hypercaloric Diet

**DOI:** 10.1155/2021/3260789

**Published:** 2021-07-27

**Authors:** Anderson Fellyp Avelino Diniz, Brena Freire de Oliveira Claudino, Manoel Vieira Duvirgens, Petruska Pessoa da Silva Souza, Paula Benvindo Ferreira, Francisco Fernandes Lacerda Júnior, Adriano Francisco Alves, Bagnólia Araújo da Silva

**Affiliations:** ^1^Postgraduate Program in Natural and Synthetic Products Bioactive/Health Sciences Center, Federal University of Paraiba, João Pessoa, Paraíba, Brazil; ^2^Health Sciences Center, Federal University of Paraiba, João Pessoa, Paraíba, Brazil; ^3^General Pathology Laboratory-Health Sciences Center-Department of Physiology and Pathology, Federal University of Paraiba, João Pessoa, Paraíba, Brazil; ^4^Pharmaceutical Sciences Department/Health Sciences Center/Federal University of Paraiba, João Pessoa, Paraíba, Brazil

## Abstract

The consumption of hypercaloric diets is related to the development of obesity, favoring the etiology of gastrointestinal disorders. In this context, *Spirulina platensis* (SP), some blue-green algae with antioxidant action, appears as a potential therapeutic alternative to prevent obesity and associated intestinal disorders. Thus, the present study is aimed at evaluating the deleterious effects of the hypercaloric diet on the contractile and relaxing reactivity of the ileum of rats, as well as the possible preventive mechanisms of dietary supplementation with SP. Wistar rats were divided into three groups: fed a standard diet (SD), a hypercaloric diet (HCD), and/or supplemented with 25 mg/kg SP (HCD + SP25) for 8 weeks. The hypercaloric diet was effective in promoting obesity in rats, as well as decreasing potency and ileal relaxing and contractile efficacy. In contrast, dietary supplementation with SP was able to prevent some of the parameters of experimental obesity. In addition, SP prevented the reduction of intestinal reactivity, possibly due to a positive modulation of voltage-gated calcium channels (Ca_V_) and negative regulation of muscarinic receptors (M3). Thus, food supplementation with *Spirulina platensis* becomes a promising alternative in the prevention of gastrointestinal diseases induced and/or aggravated by obesity.

## 1. Introduction

Defined as the abnormal and/or excessive deposition of body fat, which directly interferes with the individual's health, obesity is a chronic noncommunicable disease caused by the energy imbalance between consumption and caloric expenditure, representing an important risk factor for development of cardiovascular diseases, type 2 diabetes mellitus, musculoskeletal disorders, and some types of cancer [1–4]. The causes can be influenced by genetic, hormonal, and environmental factors, especially those related to poor eating habits, determined by the consumption of hypercaloric diets leading to increased rates of obesity and overweight and consequently to the development of various gastrointestinal disorders [5, 6].

Thus, with the growing global obesity epidemic, researchers have turned their attention to studies that demonstrate the relationship between obesity and the main metabolic and endocrine disorders that affect the gastrointestinal system (gastroesophageal reflux disease, dyspepsia, constipation, bowel syndrome irritable, and diarrhea) that contribute to the existence of similar proinflammatory mechanisms, linking both diseases [7–9]. In addition, preclinical studies report that obesity impairs inhibitory neuromuscular transmission and relaxation of enteric smooth muscle, in addition to changes in intestinal motility [10].

In this context, the marine environment represents a rich therapeutic arsenal composed of several organisms that function as sources of bioactive metabolites, with great potential for the discovery of new drugs [11]. Among these organisms, in recent years as previous marine algae gaining prominence, being increasingly inserted in human food, demonstrating antiobesity effects, mainly to its nutritional composition of bioactive compounds, photosynthetic pigments, sterols, polyunsaturated fatty acids, vitamins, minerals, fiber, and proteins, used as nutraceuticals and/or food supplements. In addition, the interest in inserting algae as one of the additives present in animal feed has been growing, mainly because they are a natural source of biomass and easily cultivated, enhancing performance and animal health [12–15].

Among the algae, *Spirulina platensis* (*Arthrospira platensis*) stands out, a blue-green, unicellular alga with nutraceutical, probiotic, antioxidant, anti-inflammatory, hypolipidemic, hypoglycemic, antihypertensive, and immunomodulatory properties [16–18]. *Spirulina* has been shown to be an efficient dietary supplement for weight control in animals and humans, due to its excellent nutritional profile and the large amount of protein elements, phycocyanin, carotenoids, and all important amino acids for the body's balance [19–22].

In recent years, our research group has studied the various effects of food supplementation with *S. platensis*, showing that the algae were able to decrease lipid peroxidation and inhibit oxidation in the aorta [23], cavernous body [24], and ileum [25] of rats, as well as the concentration of reactive oxygen species and the inflammation induced by the exercise of force [26]. In addition to reducing adipose reserves and restoring intestinal contractile reactivity in Wistar rats fed a high-fat diet for 16 weeks [27].

Therefore, it is important to evaluate the functional changes and the deleterious effects caused by the consumption of the high calorie diet on parameters related to experimental obesity and intestinal reactivity and the possible therapeutic role of food supplementation with *S. platensis* in preventing changes in reactivity relaxing and contractile ileum of Wistar rats fed for eight weeks on a calorie-rich diet, investigating the possible mechanisms of action involved in such effects.

## 2. Materials and Methods

### 2.1. Chemicals

Potassium chloride (KCl), calcium chloride (CaCl_2_), magnesium chloride (MgCl_2_), sodium chloride (NaCl), and formaldehyde were purchased from Vetec Química Fina Ltda. (João Pessoa, Brazil). Sodium bicarbonate (NaHCO_3_) and glucose (C_6_H_12_O_6_) were purchased from Dinâmica (Brazil). Sodium monobasic phosphate (NaH_2_PO_4_), sodium hydroxide (NaOH), and hydrochloric acid (HCl) were purchased from Nuclear (Brazil). These substances, except glucose, NaCl, and NaHCO_3_, were diluted in distilled water to obtain each solution, which were maintained under refrigeration.

Carbamylcholine hydrochloride (CCh) was purchased from Merck (USA). Cremophor®, thiobarbituric acid, tetramethoxypropane, perchloric acid, Mayer's hematoxylin, and eosin were acquired from Sigma-Aldrich (Brazil). All substances were diluted in distilled water as needed for each experimental protocol. The carbogen mixture (95% O_2_ and 5% CO_2_) was obtained from White Martins (Brazil).

### 2.2. Animals

Wistar male rats (Rattus norvegicus), 2 months old (approximately 170 g), were obtained from the Animal Production Unit (UPA) of the Research Institute for Drugs and Medicines, João Pessoa, Brazil (IpeFarM/UFPB). The animals were maintained under controlled ventilation and temperature (21 ± 1°C) with water ad libitum in a 12 h light-dark cycle (light on from 6 to 18 h). The experimental procedures were performed following the principles of guidelines for the ethical use of animals in applied etiology studies [28] and from the Conselho Nacional de Controle de Experimentação Animal of Brazil [29] and were previously approved by the Ethics Committee on Animal Use of UFPB with certificate number 6061090318.

The animals were randomly divided into three groups (8 rats/group): rats given a standard diet (SD), rats fed a hypercaloric diet (HCD), and fed a hypercaloric diet and simultaneously supplemented with 25 mg/kg Spirulina platensis for 8 weeks (HCD + SP25). The experimental groups were fed for 8 weeks. After this period, the animals were anesthetized with thiopental sodium (100 mg/kg body weight) mixed with lidocaine (10 mg/mL), then euthanized by decapitated by guillotine.

### 2.3. Preparation and Supplementation with Spirulina platensis

*Spirulina platensis* (Arthrospira platensis) was obtained from the INFINITY Pharma laboratory (HONG KONG, China) (lot No. 20130320), in powder form, and a sample was analyzed, fractionated, and distributed by the Roval Manipulation Pharmacy (João Pessoa, Paraíba, Brazil) (lot No. 405894) to certify that S. platensis lyophilized powder has been obtained.

The S. platensis powder was dissolved in saline solution (NaCl 0.9%) to prepare the dose of 25 mg/kg. The groups supplemented with 25 mg/kg received the supplementation for a period of 8 weeks [30]. Oral administration was done daily through stainless steel needles for gavage and 5 mL syringes with a precision of 0.2 mL, between 12 and 14 hours.

### 2.4. Food Diets

The standard diet (Presence®) contains 23% protein, 63% carbohydrate, and 4% lipids with 3.8 kcal energy density/g; the hypercaloric diet consisted of standard diet (Presence®), milk chocolate, peanuts, and sweet biscuits at a ratio of 3 : 2 : 2 : 1 [31]. The hypercaloric diet contains 23% protein, 45% carbohydrate, and 16% lipids with energy density 4.17 kcal/100 g by weight; this diet was prepared weekly and fed to the animals as granules [32, 33] (Table 1). To prepare the hypercaloric diet, the feed, peanuts, and biscuits were ground and mixed, and the chocolate melted in a water bath and added to the mixture to form a homogeneous material that was molded and then dried in an oven (70°C) for 24 h and stored at room temperature (Table 1). The diet was prepared weekly and offered to rats in the form of pellets [30].

### 2.5. Ileum Isolation

All animals were fasted for 12 hours before being euthanized. After this period, the rats were anesthetized with ketamine 100 mg/kg (i.p.) and xylazine 10 mg/kg (i.p.), and the euthanasia process was completed by decapitation in a guillotine. Then, an abdominal incision was made, and the ileum segment was removed and placed in a Petri dish containing the Krebs Henseleit solution at 37°C under aeration with carbogen.

Record isometric contractions, the adjacent connective and adipose tissues of the ileum were removed, and ileum segments (2-3 cm) were suspended in bath tubs for isolated organs (6 mL) containing Krebs Henseleit solution aerated with carbogen at 37°C and remained at rest under tension of 1 g for 30 minutes, the time necessary for the stabilization of the organ. During the stabilization period, the nutrient solution was changed every 10 minutes to avoid the interference of metabolites [34].

The physiological solution of Tyrode was used and has the composition as follows (in mM): NaCl (150.0), KCl (2.7), CaCl_2_ (1.8), MgCl_2_ (2.0), NaHCO_3_ (12.0), NaH_2_PO_4_ (0.4), and D-glucose (5.5). The pH was adjusted to 7.4, and the ileum was stabilized for 1 h under a resting tension of 1 g at 37°C and bubbled with a carbogen mixture [33, 34].

### 2.6. Parameters of Experimental Obesity

#### 2.6.1. Murinometric Parameters

On the day of euthanasia, the rats were weighed, and the nasoanal length (cm) was used, which was used to calculate the Lee index, from the ratio between the cube root of body mass (g) and the nasoanal length (cm) of the animal [35]. The body mass index (BMI) was calculated from the ratio between body mass (g) and the square of body length (cm^2^) [36]. The abdominal circumference, located in the anterior part of the animal's rear paw, and the thoracic circumference, located in the posterior portion of the front paw, were measured using an anthropometric body measuring tape [36].

#### 2.6.2. Mass of Adipose Tissue Deposits

Twenty-four hours after the last exposure of the diet and supplementation, the rats were euthanized by guillotine, and after careful dissection, the epididymal, retroperitoneal, and inguinal adipose tissues were weighed, which represent the main components of central adiposity in rats [37]. The abdominal fat located in the lower abdomen connected to the epididymis represents the epididymal fat. The fat connected to the posterior abdominal wall around the kidneys and the abdominal part of the ureter represents the retroperitoneal fat. The subcutaneous fat that is located between the lower portion of the rib cage and the median portion of the thigh represents the inguinal fat [38, 39].

#### 2.6.3. Adiposity Index

The adiposity index was calculated from the sum of the individual masses of the epididymal, retroperitoneal, and inguinal fat layers, using the following formula: (epididymal fat + retroperitoneal fat + inguinal fat) × 100/final body mass [40, 41].

### 2.7. Relaxation Reactivity Measurement

After the stabilization period, a contraction was induced with 25 mM KCl to check the organ's functionality. Then, the preparation was washed, and a new contraction was induced with 25 mM KCl, and under the tonic component of this contraction was added verapamil (10^−16^ to 3 × 10^−7^ M), a blocker of calcium channels dependent on voltage, cumulatively to the vat, in all preparations [42]. The results were evaluated by comparing the responses of the control groups supplemented with saline, obese supplemented with saline, and obese supplemented with S. platensis (25 mg/kg), and the values of *E*_max_ and pCE_50_ were evaluated as parameters of efficacy and potency, respectively. The values of *E*_max_ and pCE_50_ were obtained by nonlinear regression.

### 2.8. Contraction Reactivity Measurement

After the stabilization period, the ileum was contracted with 25 mM KCl to check the integrity of the organ; then, the organ was washed, and after returning to baseline, atropine was preincubated (10^−6^ and 10^−5^) [43, 44], a nonselective antagonist of muscarinic receptors. Subsequently, a cumulative replenished concentration curve for carbacol, an analogue of acetylcholine, was performed. The results were evaluated by comparing the responses of the control groups supplemented with saline (SD and HCD) and supplemented with S. platensis (HCD + SP25), and the values of *E*_max_ and pCE_50_ were evaluated as parameters of efficacy and potency, respectively.

### 2.9. Ileal Histological and Morphometric Analysis

For a general analysis of the histoarchitecture of the analyzed tissues, the samples were placed in an automatic tissue processor, embedded in paraffin and then cut in a 4 *μ*m microtome, placed on histological slides and deparaffinized in xylene for 30 minutes, hydrated in alcohols in decreasing concentrations for 25 minutes and washed in running water for 5 minutes, and then washed in distilled water. The samples were then treated with Harris hematoxylin for 1 minute, washed again in distilled water for 5 minutes and again stained with eosin for 3 minutes, and later washed in running water for another 30 seconds. Finally, the slides were dehydrated in increasing concentrations of alcohols, cleared in xylene, and mounted with Entellan®.

## 3. Statistical Analysis

The results were expressed as mean and standard error of the mean, being analyzed statistically using the *t*-test (unpaired) or the analysis of variance (ANOVA) one way followed by the Tukey posttest. The null hypothesis was rejected when *p* value < 0.05. As a power parameter, pCE_50_ values were used; calculated by nonlinear regression and as an efficiency parameter, *E*_max_ was used [45]. All data were analyzed using the GraphPad Prism® program version 6.01 (GraphPad Software Inc., San Diego, CA, USA).

## 4. Results

### 4.1. Effect of Hypercaloric Diet and Supplementation with *S. platensis* on Final Body Mass and Murinometric Parameters

**Figure 1:**
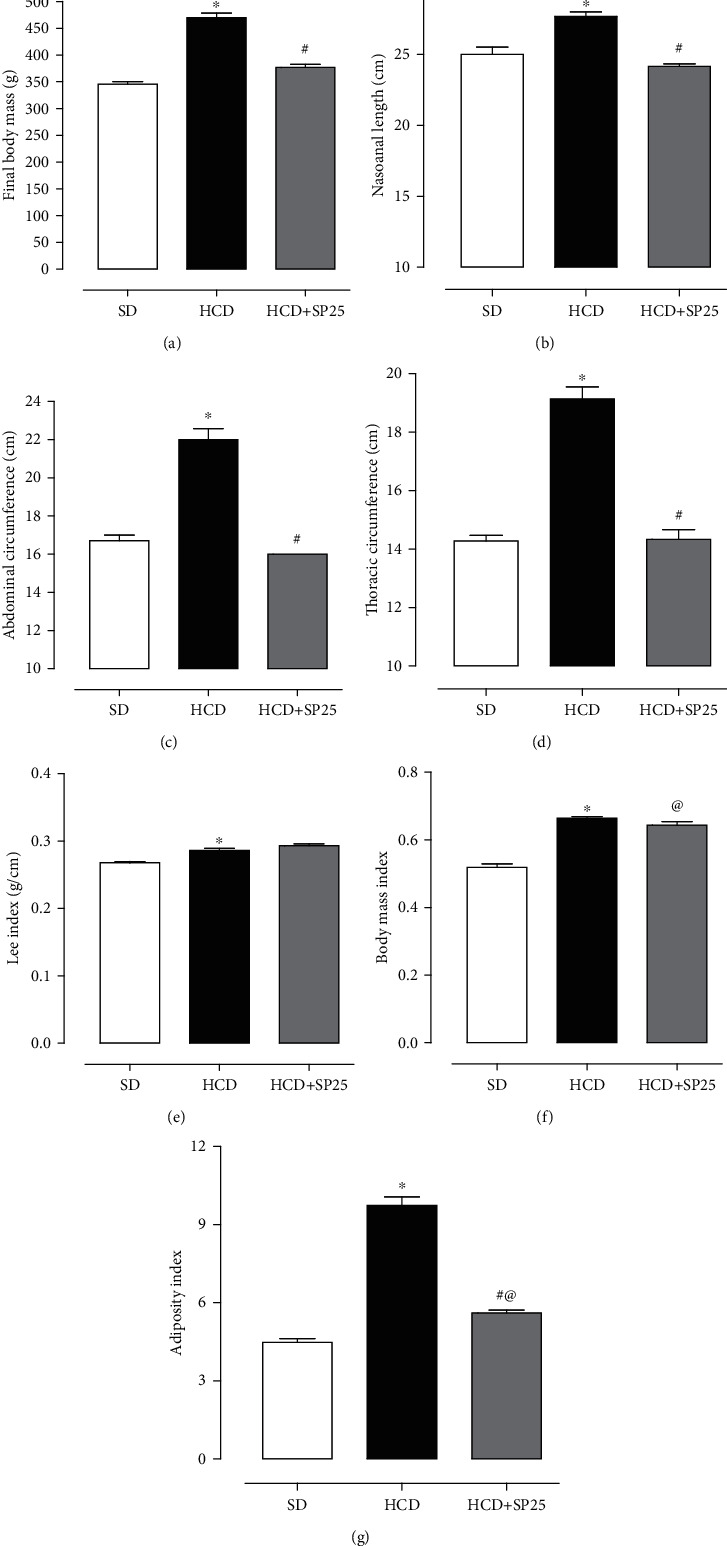
Values of final body mass (a), nasoanal length (b), waist circumference (c), chest circumference (d), Lee index (e), body mass index (f), and adiposity index (g) in rats from group DS, HCD, and HCD + SP25. Columns and vertical bars represent the mean and S.E.M., respectively (*n* = 5). ANOVA was one-way followed by Tukey's posttest. ^∗^*p* < 0.05 (SD vs. HCD); ^#^*p* < 0.05 (HCD vs. HCD + SP25); ^@^*p* < 0.05 (SD vs. HCD + SP25). SD: standard diet group supplemented with saline; HCD: hypercaloric diet group supplemented with saline; HCD + SP25: hypercaloric diet group and supplemented with *S. platensis* at a dose of 25 mg/kg.

In rats fed the hypercaloric diet, an increase in final body mass (470.1 ± 8.2 g) was observed when compared to rats fed the standard diet (345.7 ± 4.7 g). The rats fed the hypercaloric diet and supplemented with *S. platensis* at a dose of 25 mg/kg (376.7 ± 5.9) showed an increase in body mass when compared to the SD group and a decrease in relation to the HCD group (Figure 1(a)).

When analyzing murinometric parameters, the DHC group had both nasoanal (27.67 ± 0.3 cm) and abdominal (22.0 ± 0.6 cm) and thoracic (19.14 ± 0.4 cm) length difference in relation to the SD group (25.0 ± 0.5 cm, 16.71 ± 0.3 cm, and 14.29 ± 0.2 cm, respectively). Analyzing the HCD + SP25 group, it had nasoanal length (24.17 ± 0.2 cm), lower abdominal (16.00 ± 0.1 cm), and thoracic (5 cm) circumferences when compared to the group HCD and with no difference with the SD group (Figures 1(b)–1(d)).

Regarding the Lee index, the HCD group (0.817 ± 0.003 g/cm) was higher than the SD (0.72 ± 0.01 g/cm) (Figure 1(e)). In the body mass index (BMI), the HCD group (0.66 ± 0.010) showed an increase in relation to the SD group (0.52 ± 0.01), but the HCD + SP25 group (0.64 ± 0.01) did not differ from the HCD group, showing a difference only when compared to the SD control group (Figure 1(f)). As for the adiposity index, both the HCD group (9.74 ± 0.33) and the HCD + SP25 group (5.61 ± 0.11) showed an increase in relation to the SD group (4.47 ± 0.15). The HCD + SP25 group, when compared to the obese group, had a lower adiposity index (5.61 ± 0.11) (Figure 1(g)).

### 4.2. Effect of Consumption of the Hypercaloric Diet and Food Supplementation with *S. platensis* on the Mass of Adipose Tissue Deposits

**Figure 2:**
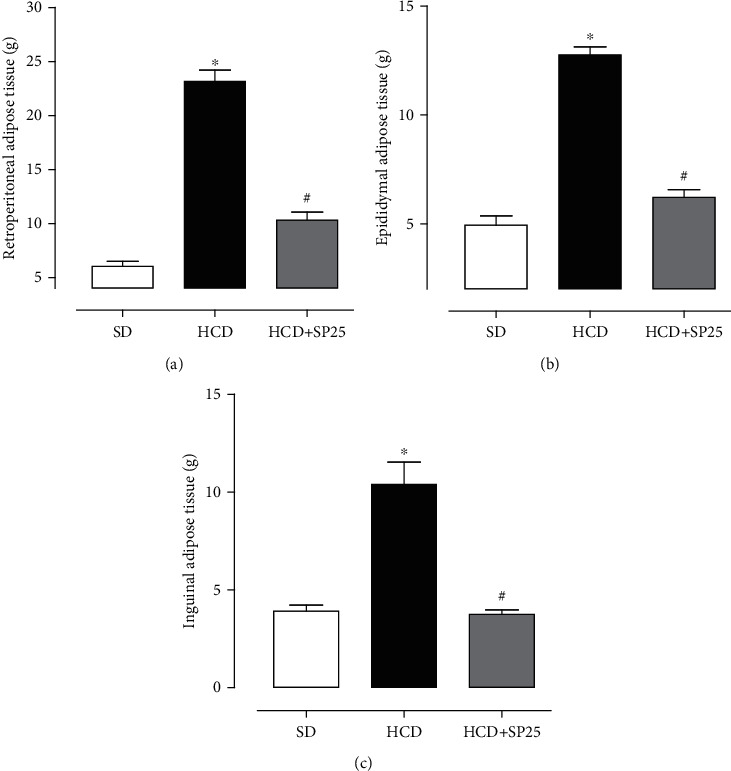
Values of the masses of adipose tissue deposits: retroperitoneal (a), epididymal (b), and inguinal (c) in rats from groups SD, HCD, and HCD + SP25. Columns and vertical bars represent the mean and S.E.M., respectively (*n* = 5). ANOVA was one-way followed by Tukey's posttest. ^∗^*p* < 0.05 (SD vs. HCD); ^#^*p* < 0.05 (HCD vs. HCD + SP25); ^@^*p* < 0.05 (SD vs. HCD + SP25), (*n* = 5). SD: standard diet group supplemented with saline; HCD: hypercaloric diet group supplemented with saline; HCD + SP25: hypercaloric diet group and supplemented with *S. platensis* at a dose of 25 mg/kg.

Analyzing the mass of adipose tissue deposits, it was possible to observe that the HCD group rats showed an increase in retroperitoneal (23.16 ± 1.0 g), epididymal (12.76 ± 0.4 g), and inguinal (10.4 ± 1.1 g) when compared to the SD group (6.05 ± 0.4 g, 4.94 ± 0.4 g, and 3.92 ± 0.3 g, respectively) (Figures 2(a)–2(c)). However, the group fed a hypercaloric diet and supplemented with 25 mg/kg S. platensis observed in the retroperitoneal adipose tissues (10.32 ± 0.7 g), epididymal (6.22 ± 0.3 g), and inguinal (13.76 ± 0.2 g) relative decrease in fat deposition when compared to the obese HCD group, with no significant difference in SD (Figures 2(a)–2(c)).

### 4.3. Effect of Consumption of the Hypercaloric Diet and Food Supplementation with *S. platensis* on the Relaxing Reactivity of Isolated Rat Ileum

**Figure 3:**
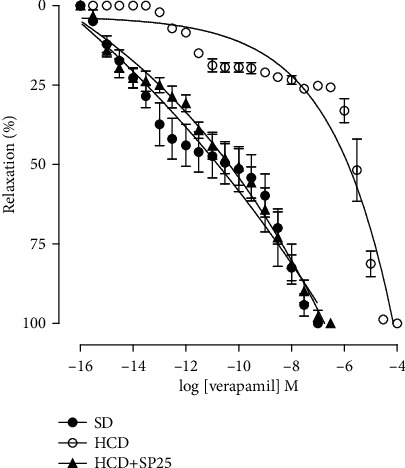
Cumulative concentration-response curve for verapamil in rat ileum in the SD (●), HCD (○), and HCD + SP25 (▲) groups. The symbols and vertical bars represent the mean and S.E.M., respectively (*n* = 5). ANOVA was one-way followed by Tukey's posttest. ^∗^*p* < 0.05 (SD vs. HCD), ^#^*p* < 0.05 (HCD vs. HCD + SP25), and ^@^*p* < 0.05 (SD vs. DHC + SP25). SD: group fed a standard diet; HCD: group fed a hypercaloric diet; HCD + SP25: groups fed a hypercaloric diet and supplemented with *S. platensis* 25 mg/kg, respectively.

In the HCD group, a decreased KCl contractile power was observed in the presence of verapamil (pCE_50_ = 6.09 ± 0.21) when compared to the group that consumed only the standard diet (pCE_50_ = 10.90 ± 0.52). In addition, it can be observed that there were no changes regarding the relaxing potency of verapamil between the HCD + SP25 group (pCE_50_ = 10.55 ± 0.33) and SD; however, it is possible to verify an increase in power when compared to the HCD group (Figure 3).

### 4.4. Effect of Consumption of the Hypercaloric Diet and Food Supplementation with *S. platensis* on the Cumulative Curve of Contraction Induced by Carbachol in the Absence and Presence of Atropine in the Groups Fed the Standard Diet

In the presence of 10^−6^ M atropine, it was observed that the cumulative contraction curve for CCh shifted to the right with an increase in pCE_50_ (3.5 ± 0.2) when compared to the contraction curve in the absence of the antagonist (6.3 ± 0.04) without changing *E*_max_ (100%). Similar results were observed in the presence of 10^−5^ M atropine, in which the CCh contraction curve was shifted to the right, with an increase in pCE_50_ (2.3 ± 0.01) without changing *E*_max_ (100%).

### 4.5. Effect of Consumption of the Hypercaloric Diet and Food Supplementation with *S. platensis* on the Cumulative Curve of Contraction Induced by Carbachol in the Absence and Presence of Atropine in the Groups Fed the Hypercaloric Diet

**Figure 4:**
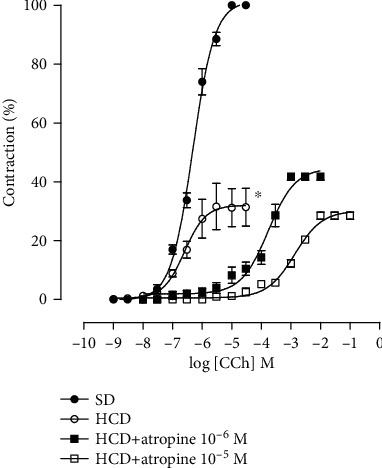
Cumulative concentration-response curves on the CCh of the SD (●), HCD (○), HCD + atropine 10^−6^ M (■), and HCD + atropine 10^−5^ M (□) groups in isolated rat ileum. The symbols and vertical bars represent the mean and S.E.M., respectively (*n* = 5). ANOVA was one-way followed by Tukey's posttest. ^∗^*p* < 0.05 (SD vs. HCD), ^#^*p* < 0.05 (HCD vs. HCD + atropine 10^−6^ M and 10^−5^ M), and ^@^*p* < 0.05 (SD vs. HCD + atropine 10^−6^ M and 10^−5^ M). SD: group fed a standard diet; HCD: group fed a hypercaloric diet; HCD + SP25: groups fed a hypercaloric diet and supplemented with *S. platensis* 25 mg/kg, respectively.

In the group fed during the eight weeks with the hypercaloric diet, a decrease in the maximum effect was observed (32.0 ± 2.9) when compared to the contraction curve of the group of animals that were fed a standard diet (6.3 ± 0.04) without changing the pCE_50_ (6.6 ± 0.01 and 6.3 ± 0.04, respectively) (Figure 4).

In the HCD group in the presence of 10^−6^ M atropine, it was observed that the cumulative contraction curve in CCh was shifted to the right with an increase in pCE_50_ (3.9 ± 0.08) when compared to the contraction curve in the absence of the antagonist in the group HCD (6.6 ± 0.01) with a change in *E*_max_ (32.0 ± 2.9 and 44.6 ± 2.0%, respectively). Similar results were observed in the presence of 10^−5^ M atropine, in which the CCh contraction curve was shifted to the right, with an increase in pCE50 (2.9 ± 0.03) with an *E*_max_ change (30.2 ± 0.9%) (Figure 4).

### 4.6. Effect of Consumption of the Hypercaloric Diet and Food Supplementation with *S. platensis* on the Cumulative Curve of Contraction Induced by Carbachol in the Absence and Presence of Atropine in the Groups Fed a Hypercaloric Diet and Supplemented Simultaneously with *S. platensis* at a Dose of 25 mg/kg

**Figure 5:**
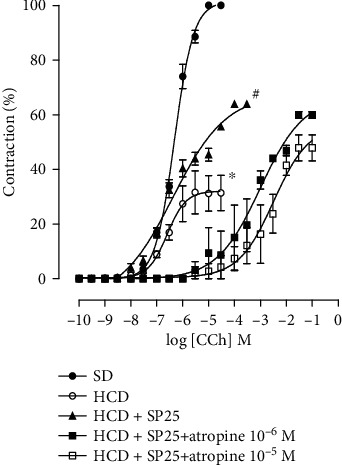
Cumulative concentration-response curves on the CCh of the SD (●), HCD (○), HCD + SP25 (▲), HCD + SP25 + atropine 10^−6^ M (■), and HCD + SP25 + atropine 10^−5^ M (□) groups in isolated rat ileum. The symbols and vertical bars represent the mean and S.E.M., respectively (*n* = 5). ANOVA was one-way followed by Tukey's posttest. ^∗^*p* < 0.05 (SD vs. HCD), ^#^*p* < 0.05 (HCD vs. HCD + SP25), and ^@^*p* < 0.05 (HCD + SP25 vs. HCD + SP25 + atropine 10^−6^ M and 10^−5^ M). SD: group fed a standard diet; HCD: group fed a hypercaloric diet; HCD + SP25: groups fed a hypercaloric diet and supplemented with *S. platensis* 25 mg/kg, respectively.

In the group submitted to a hypercaloric diet and supplemented with *Spirulina platensis* at a dose of 25 mg/kg (HCD + SP25) in the presence of 10^−6^ M atropine, it was observed that the cumulative contraction curve shifted to the right with decreased potency (pCE_50_ = 3.4 ± 0.4) when compared to the contraction curve in the absence of the antagonist in the SD group (pCE_50_ = 6.3 ± 0.04) with a change in (*E*_max_ = 59.89 ± 1.2 and 100%, respectively); similar results occurred when compared to the contraction curve in the absence of the antagonist in the HCD group (pCE_50_ = 6.6 ± 0.1) with change in the maximum effect (*E*_max_ = 59.89 ± 1.2 and 32.0 ± 2.9%, respectively) and when compared to the contraction curve in the absence of the antagonist in the HCD + SP25 group (pCE_50_ = 6.2 ± 0.1) with change in the maximum effect (*E*_max_ = 59.89 ± 1.2 and 63.87 ± 1.0%, respectively) (Figure 5).

Similar results were observed in the group submitted to a hypercaloric diet and supplemented with *Spirulina platensis* at a dose of 25 mg/kg (HCD + SP25) in the presence of 10^−5^ M atropine, in which the CCh contraction curve was shifted to right with decreased contractile force (pCE_50_ = 2.8 ± 0.3) with a change in the maximum effect (*E*_max_ = 47.93 ± 4.8%) (Figure 5).

### 4.7. Effect of Hypercaloric Diet and *Spirulina platensis* Supplementation on Histology and Ileal Morphometry of Rats

**Figure 6:**
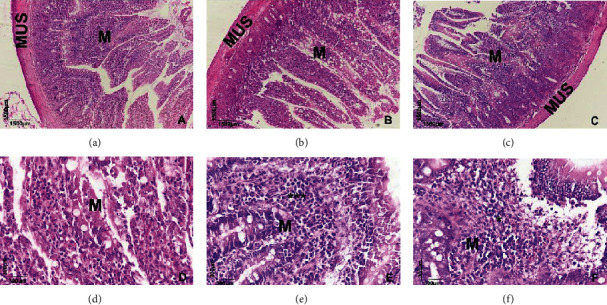
Histological section of rat ileum stained in hematoxylin and eosin showing parts of the organ, highlighting the mucosa (M) and the external muscle (MUS). Panoramic view (a–c) and higher magnification (d–f) showing histological images of the ileum of rats from SD, HCD, and HCD + SP25 groups, respectively.

In animals in the SD group, it is observed that the mucosa has some mononuclear inflammatory cells, such as macrophages and few plasmocytes, with preservation of goblet cells and enterocytes (Figures 6(a) and 6(d)). In animals in the HCD group, it is observed that the inflammatory infiltrate in the mucosa is extensive and multifocal (∗∗∗), rich in mononuclear cells, such as macrophages and many plasmocytes (Figures 6(b) and 6(e)). In animals from the HCD-SP25 group, a slight increase in the number of mononuclear cells (∗), with the same phenotype, macrophages, and lymphocytes, is observed in the mucosa (Figures 6(c) and 6(f)).

**Figure 7:**
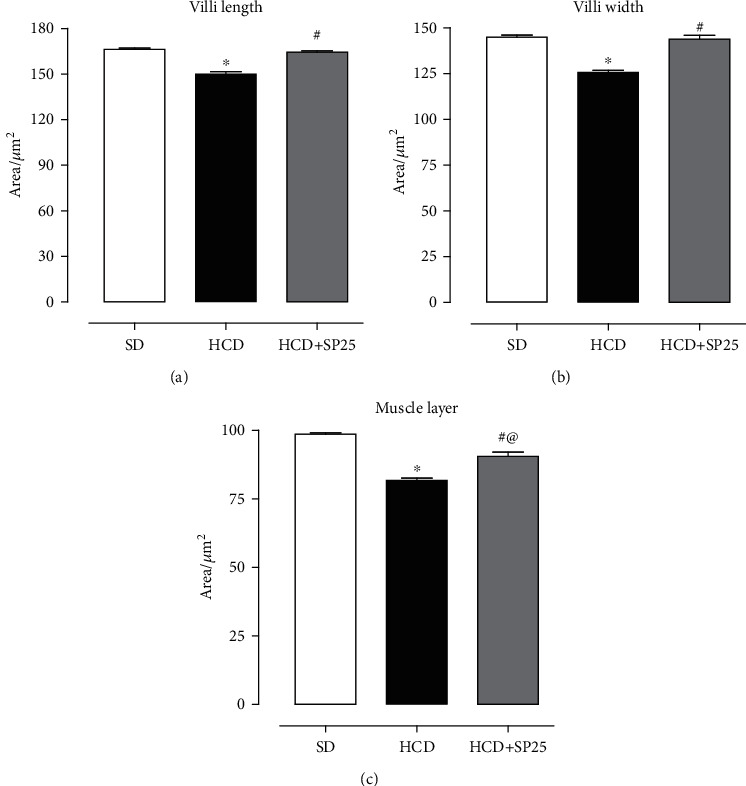
Ileum morphometry showing villi length (a), villi width (b), and muscle layer (c) of groups the SD, HCD, and HCD + SP25. Columns and vertical bars represent the mean and S.E.M., respectively (*n* = 5). ANOVA was one-way followed by Tukey's posttest. ^∗^*p* < 0.05 (SD vs. HCD), ^#^*p* < 0.05 (HCD vs. HCD + SP25), and ^@^*p* < 0.05 (HCD + SP25 vs. HCD + SP25). SD: group fed a standard diet; HCD: group fed a hypercaloric diet; HCD + SP25: groups fed a hypercaloric diet and supplemented with *S. platensis* 25 mg/kg, respectively.

In the ileal morphometric analysis of the mucosal area, it was observed that the animals in the HCD group (149.9 ± 1.6) had a decrease in the length of the intestinal villi when compared to the SD control group (166.3 ± 0.8). The HCD + SP25 group (164.4 ± 0.8) showed no difference compared to the SD group (Figure 7(a)). Similarly, when analyzing the width of the intestinal villi of the ileum of rats in the HCD group (125.6 ± 1.2), a decrease was observed in relation to the SD group (145 ± 1.0). The group supplemented with *Spirulina* at a dose of 25 mg/kg (143.±2.0) also showed no difference when compared to the control group (145 ± 1.0) (Figure 7(b)).

Likewise, in the morphometric analysis of the outer muscle layer, it was observed that the group fed the high-calorie diet HCD (81.7 ± 0.9) presented a decrease in the muscle layer compared to the SD group (98.5 ± 0.4). Interestingly, the ileal muscle layer of the HCD + SP25 group (90.5 ± 1.5) presented differences in relation to both the HCD (81.7 ± 0.9) and SD (98.5 ± 0.4) groups (Figure 7(c)).

## 5. Discussion

In the present study, the experimental obesity model was induced by consuming a hypercaloric diet in Wistar rats for 8 weeks, which resulted in increased final body mass, murinometric parameters, and body adiposity index, as well as promoting a reduction in relaxing and ileal contractile reactivity. Interestingly, the deleterious effects promoted by the consumption of the hypercaloric diet were prevented by supplementing food with *S. platensis*.

Obesity for being able to cause comorbidities in individuals is already considered a serious public health problem [46]. Thus, to better understand the effects and mechanisms that the disease brings to, the individual, hypercaloric diets are used to induce a state of experimental obesity in rats, mimicking the consequences that they would bring to humans [47]. Wistar rats were fed a standard and/or hypercaloric diet for eight weeks and simultaneously received saline solution and/or supplementation with *S. platensis* at a dose of 25 mg/kg/day, based on the principle that in previous studies, carried out by our research group, this was the dose that had the best effects [48].

When analyzing the data related to experimental obesity, it is possible to observe that the rats fed with the hypercaloric diet had an increase in the final body mass and that the supplementation with *Spirulina platensis* at a dose of 25 mg/kg totally prevented this increase, not having a significant difference when compared to the control group fed a standard diet (Figure 1(a)). Such results suggest that the consumption of the hypercaloric diet was effective in increasing the final body mass, consequently favoring the onset of obesity in these animals. In addition, the protective effect attributed to kelp can be related to its composition and properties, characterized by a high content of proteins (approximately 70%), bioactive compounds, and antioxidants, which contribute to the thermogenesis process and decrease lipogenesis, preventing therefore, the accumulation of lipid and the deposition of fat from consumption of the hypercaloric diet [49, 50].

To verify the effectiveness of the hypercaloric diet in the development of obesity as well as the possible protective role of supplementation with *S. platensis*, parameters related to experimental obesity were evaluated (Lee index, BMI, adiposity, nasoanal length, measured abdominal and thoracic circumference, and mass of fat reserves). From these results, it is possible to infer the evidence of the onset of obesity from the hypercaloric diet used in this study. Additionally, dietary supplementation with algae at a dose of 25 mg/kg was effective in partially and/or totally preventing some of the analyzed parameters (Figures 1(b)–1(d) and 1(g)), thus reaffirming its antiobesity effects. However, no differences were observed between the Lee index and the BMI of the experimental groups (Figures 1(e) and 1(f)), similar to that observed in different studies with animal obesity induced by excessive intake of sucrose (300 g/L) or lipids (42.9% lipids per kcal) [51, 52].

In animals, mainly rats, fat deposition occurs predominantly in three distinct regions; these reserves are located in the retroperitoneal, epididymal, and inguinal adipose tissues. The assessment of the mass of these regions helps to determine the physiological dysfunctions resulting from the ingestion of hyperlipidic and/or hypercaloric diets [53–55]. Thus, it was observed that the obese rats showed an increase in adipose, retroperitoneal (Figure 2(a)), epididymal (Figure 2(b)), and inguinal (Figure 2(c)) deposits and that food supplementation with *S. platensis* was able to prevent this increase in all of them. Furthermore, based on these results, a possible modulating activity of *S. platensis* in preventing and reducing adipose deposits of rats fed a hypercaloric diet is demonstrated.

Once the development of obesity and the physiological changes caused by the consumption of the hypercaloric diet were confirmed and that supplementation with *S. platensis* prevented such dysfunction, the investigation of the impact of diet and supplementation with algae on relaxing reactivity was continued intestinal, since the increase in body adiposity is associated with decreased relaxing reactivity in ileum of Wistar rats, and food supplementation with *S. platensis* at a dose of 50 mg/kg was able to reverse these changes [27].

**Figure 8:**
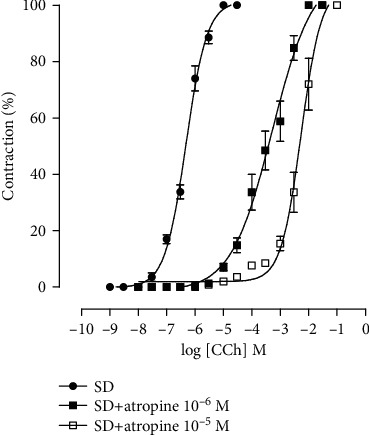
Cumulative concentration-response curves for the CCh of the SD (●), SD + atropine 10^−6^ M (■), and SD + atropine 10^−5^ M (□) groups in isolated rat ileum. The symbols and vertical bars represent the mean and S.E.M., respectively (*n* = 5). ANOVA was one-way followed by Tukey's posttest. ^∗^*p* < 0.05 (SD vs. HCD), ^#^*p* < 0.05 (HCD vs. HCD + SP25), and ^@^*p* < 0.05 (SD vs. HCD + SP25). SD: group fed a standard diet; HCD: group fed a hypercaloric diet; HCD + SP25: groups fed a hypercaloric diet and supplemented with *S. platensis* 25 mg/kg, respectively.

In this study, it was demonstrated that the hypercaloric diet decreased the ileal relaxing reactivity, confirmed by the decrease in the relaxing efficacy of verapamil (Figure 8), making this relaxation difficult. In addition, supplementation with *S. platensis* completely prevented the decrease in both the potency and the relaxing effectiveness of the electromechanical component, bringing them back to what was observed in the control group, confirmed by the overlapping curves. Likewise, the hypercaloric diet reduced the contractile effectiveness of KCl, proving that it reduces the contractions caused by electromechanical coupling [33]. In contrast, Ferreira [27] did not observe any difference between animals fed a standard diet supplemented with *S. platensis* in relation to the obese group, and it is not possible to observe a reversal of such effects. However, our study demonstrated that food supplementation with *S. platensis* at a dose of 25 mg/kg prevented changes caused by the high calorie diet, thus inferring that algae may be a possible alternative for the prevention of intestinal diseases in obese people.

Studies have shown that obesity increases vasoconstriction in the aorta of obese rats, in which possibly the excessive accumulation of adipose tissue is related to increasing the expression of proteins in the calcium sensitization pathway [56] and therefore decreasing the potency of ileal relaxants, and it may be suggested that this effect is associated with a decrease in the expression of voltage-gated calcium channels (Ca_V_) [57, 58]. Consequently, *S. platensis* may be preventing the decrease in calcium influx, improving changes in intestinal reactivity. Thus, to validate this hypothesis, there is a need for investigative studies on the participation of these channels in the ileum of obese rats supplemented with *S. platensis*.

Once all these deleterious damages resulting from the consumption of the hypercaloric diet were verified, and as in previous studies, it was observed that the consumption of this diet is directly related to the increase in contractile reactivity and reduction in the relaxing response of various organs in rats [24, 59, 60]; it was decided to investigate the mechanism of action by which the ingestion of the hypercaloric diet promotes a reduction in the contractile reactivity of the ileum [48], effects that were prevented in rats fed the hypercaloric diet by supplemental feeding with *S. platensis*.

The intestinal contractile reactivity was evaluated using as a premise the property that the intestinal smooth muscle has to undergo actions mediated by acetylcholine (ACh), which when it binds to its muscarinic receptor type M_3_, highly expressed in this musculature, triggers contractile responses [61]. Thus, to mimic the pharmacological action of this hormone, carbachol, an agonist of ACh muscarinic receptors, resistant to degradation by acetylcholinesterase, was used to assess the pharmacomechanical coupling of ileum contraction in Wistar rats [62].

Recently, Souza et al. [33] found that Wistar rats fed for 8 weeks on a hypercaloric diet showed reduced intestinal contractile efficacy compared to KCl and CCh. Thus, once the onset of obesity was confirmed and a reduction in ileal contractile reactivity was verified, the hypothesis was raised that the consumption of the hypercaloric diet could decrease the contractile response to CCh by negatively modulating the muscarinic receptors present in the intestinal smooth muscle and that by its antioxidant role, supplementation could prevent this modulation by possibly acting on calcium influx.

For this, in the group fed a standard diet, atropine (10^−6^ and 10^−5^ M), an antagonist of muscarinic receptors, was incubated, showing that in both concentrations the cumulative contraction curve to CCh was shifted to the right with decreased force and no change in the maximum effect (Figure 8). These results were already expected, since atropine is a competitive antagonist of muscarinic receptors, requiring increasing concentrations of carbachol to displace it from the M_3_ receptor in the ileum to obtain the same contractile effect [63]. However, this protocol is of fundamental importance for the analysis and comparison of the effects and potencies of the maximum amplitudes of the HCD and HCD + SP25 mg/kg groups with those of the control group, fed only with a standard diet. It is also possible to observe that the presence of atropine in both concentrations continued to shift the contraction curve to the right without changing the maximum effect, suggesting, therefore, that obesity and/or the hypercaloric diet did not alter the expression of muscarinic receptors, per se.

In this study, it was demonstrated that the consumption of a hypercaloric diet significantly decreased the ileal contractile response to CCh. In the HCD group in the presence of 10^−6^ and 10^−5^ M atropine, it was observed that the cumulative contraction curves of the CCh shifted to the right with decreased contractile force, with a change in the maximum effect (Figure 4). This decrease in ileal contractile reactivity does not involve the participation of muscarinic receptors, possibly being associated with modulation of the Ca_V_ and/or related to mechanisms involving the antioxidant system, mainly due to the increased expression of malondialdehyde (MDA), the main product of lipid peroxidation, corroborating the findings of previous studies by our research group [27, 33].

Previous studies carried out by our research group showed that food supplementation with the alga S. platensis was able to improve contractile reactivity in the aorta, avoiding oxidative stress [23] and restoring erectile function in obese rats [24], as well as is related to increased contractile reactivity in the ileum of rats fed a hypercaloric diet [25] by promoting increased bioavailability of nitric oxide.

Additionally, excessive consumption of fats and calories is associated with metabolic and gastrointestinal disorders. Furthermore, animal research has shown that long-term consumption of high-calorie diets is particularly related to damage to the gastrointestinal mucosa [64, 65]. Thus, intestinal histological and morphometric parameters are very important and used as scientific tools to evidence such findings and particularly the effects of the hypercaloric diet as well as *Spirulina platensis* on the histomorphology of the ileum of rats have not yet been elucidated.

The intestinal mucosal barrier plays essential roles in preserving intestinal health [66]. Among the components of the intestinal epithelial mucus layer is mucin, synthesized and secreted by goblet cells; this mucus layer is responsible for separating part of the intestinal content from the intestinal epithelial cells, protecting the cells against the invasion of harmful substances and has an active role in regulating mucosal immunity [67–69]. Our results showed that the consumption of the high-calorie diet considerably reduced the number of goblet cells and enterocytes (Figures 6(b) and 6(e)) important for digestion and absorption of nutrients from the diet, which is associated with changes in the intestinal permeability of the intestinal epithelial cell barrier, inducing oxidative stress and apoptosis. Furthermore, the high-calorie diet considerably increased the inflammatory profile of the ileum (Figures 6(b) and 6(e)) observed in the images due to the rich presence of mononuclear cells, specifically macrophages and plasma cells, characteristic of the body's adipose tissue accumulation process (D7, D8). Morphometric analyses of the intestinal mucosa of the ileum confirm the histological results, since it is possible to observe that the length (Figure 7(a)) and width (Figure 7(b)) of the ileum intestinal villi as well as the muscle layer (Figure 7(c)) presented a decrease in relation to the control group.

On the other hand, *Spirulina platensis* was able to prevent the harmful effects on the ileum mucous layer caused by the consumption of the high-calorie diet, approaching the histological findings of the control group, characterized by a slight increase in the number of mononuclear cells of the same phenotype and number of reduced macrophages and lymphocytes (Figures 6(c) and 6(f)). This effect may be associated with the nutritional composition of the alga, composed of high levels of antioxidants, carotenoids, phycocyanins, and vitamins, in addition to the pharmacological and biological activities already evidenced, which are related to gastropopotetic activities, anti-inflammatory, and antioxidant effects, in addition to increased antioxidant capacity of various organs [17, 68–70], which was confirmed by the morphometric analysis of the ileal mucosa in which all parameters evaluated were preserved (Figure 7) and did not distinguish from the healthy control group shown in Figure 7.

In view of these results, it is possible to evidence the effectiveness of the experimental obesity model in promoting damage on the intestinal reactivity of the ileum of Wistar rats and that the supplementation with *Spirulina platensis* at a dose of 25 mg/kg was potentially capable of preventing the deleterious effects induced by the consumption of hypercaloric diet during the 8-week period. In addition, this research demonstrated the beneficial potential of dietary supplementation with *S. platensis* in obesity, as well as in the prevention of intestinal disorders associated with reactivity of intestinal smooth muscle, such as diarrhea, constipation, and poor digestion, which may be associated with improvement and prevention of intestinal dysbiosis, very associated with obesity and intake of irregular diets. In view of the popularity of *Spirulina platensis* consumption as a functional food and its safety already proven, the results of this research and previous works in the literature justify the execution of clinical trials to investigate the possible protective role of this blue-green algae in diseases and intestinal disorders associated with obesity and other inflammatory pathologies.

## 6. Conclusion

**Figure 9:**
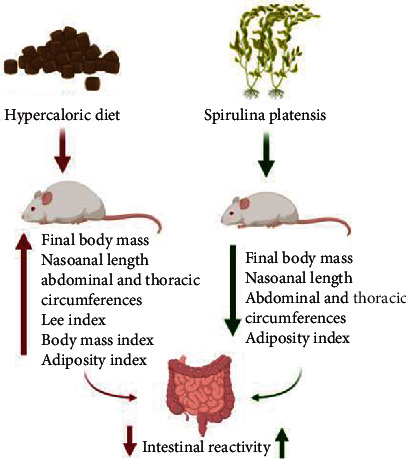
The consumption of the hypercaloric diet induces the onset of obesity in rats by increasing all parameters evaluated in this study and consequently leads to a decrease in ileal reactivity. Food supplementation with *Spirulina platensis* prevents the development of obesity and the reduction of contractile reactives and intestinal relaxants.

Food supplementation with the algae *Spirulina platensis*, the research star of this study, points to a possible preventive antiobesity role and a protector of deleterious effects and preserving the intestinal histomorphological environment on intestinal reactivity (Figure 9). In addition, the hypercaloric diet decreases contractile reactivity in rat ileum by mechanisms that are related to a downregulation of muscarinic receptors, and dietary supplementation with algae prevents the reduction of intestinal reactivity induced by the consumption of the hypercaloric diet, possibly by modulating the expression positively of the Ca_V_. These data suggest the beneficial effects of algae as a potential therapeutic arsenal in the prevention of obesity and associated intestinal diseases, diarrhea, irritable bowel disease, and some types of gastrointestinal cancer.

## Figures and Tables

**Table 1:** Macronutrients and caloric value of standard and hypercaloric diets.

Diets	Carbohydrates (%)	Proteins (%)	Lipids (%)	Total energy value (kcal/100 g)
Standard	63	23	4	3800
Hypercaloric	45.53	22.76	16	4170

Souza et al. [32].

## Data Availability

The hypothesis and review data used to support the findings of this study are included within the article.
